# Elective transfemoral amputation and simultaneous implantation of a transcutaneous osseointegrated prosthesis stem as salvage treatment after knee joint arthrodesis with poor function: A case report

**DOI:** 10.3389/fsurg.2022.918303

**Published:** 2022-08-30

**Authors:** Katharina Krause, Katherina Richter, Thomas Beyer, Horst Heinrich Aschoff, Dagmar-Christiane Fischer, Thomas Mittlmeier

**Affiliations:** ^1^ Department of Traumatology, Hand and Reconstructive Surgery, University Medical Centre Rostock, Rostock, Germany; ^2^ Department of Pediatrics, University Medical Centre Rostock, Rostock, Germany; ^3^ Department of Diagnostic and Interventional Radiology, Pediatric Radiology and Neuroradiology, University Medical Centre Rostock, Rostock, Germany

**Keywords:** ACL reconstruction, surgical complication, knee joint arthrodesis, transfemoral amputation, transcutaneous osseointegrated prosthesis system, gait analysis

## Abstract

**Background:**

Surgical reconstruction of anterior cruciate ligament ruptures is a well-established procedure, and although it is for the vast majority of patients without severe complications, total knee joint arthroplasty, arthrodesis of the knee, and finally transfemoral amputation have to be considered in the worst-case scenario.

**The case:**

We report a case of a patient with a 13-year history of recurrent failure after anterior cruciate ligament reconstruction. She claimed she had severely impaired mobility secondary to a knee joint arthrodesis *via* an Ilizarov circular frame 2 years ago and chronic immobilizing pain, making a permanent medication with opioids necessary. She was aware of the therapeutic options and asked for transfemoral amputation and concomitant supply with a transcutaneous osseointegrated prosthesis system (TOPS).

**Procedures:**

After careful evaluation and clinical work-up, the indication for transfemoral amputation and concomitant implantation of the prosthetic stem into the femoral cavity was secured. Six weeks after the creation of the stoma for coupling of the artificial limb and onset of physiotherapy, balance and gait training were scheduled. Full weight-bearing and walking without crutches were allowed 12 weeks after the index procedure. This sequence of events was paralleled by a series of pre-defined examinations, that is, questionnaires and mobility scores addressing the situation of transfemoral amputees, as well as standardized clinical gait analysis. The latter was performed before surgery and 6, 9, and 18 months after the index procedure.

**Outcome:**

At the time of the index procedure, opioids could be tapered to zero, and the patient quickly regained her walking abilities during the rehabilitation period. Clinical gait analysis confirmed the restoration of bilateral symmetry by mutual approximation of kinematics and kinetics to a standard gait pattern.

**Conclusion:**

The outcome of our patient strengthens the therapeutic potential of a unilateral transfemoral amputation in combination with TOPS. Nevertheless, long-term follow-up is necessary to detect future complications of this approach.

## Introduction

Anterior cruciate ligament (ACL) ruptures are among the most common knee injuries, and surgical reconstruction generally allows for return to pre-injury level of activity in 75%–97% of patients (1). However, failure of the procedure and severe complications such as persistent functional impairments, osteoarthritis, chronic pain, and deep infections may occur in a reasonable number of patients (1). In the worst-case scenario, total knee joint arthroplasty, arthrodesis of the knee, and transfemoral amputation have to be considered as salvage procedures (2, 3). Although the latter option is irreversible by nature, it has the potential to restore functional abilities if performed properly, that is, surgery and fitting with an artificial leg (4). In general, coupling between the artificial leg and the residual limb is achieved by the use of an individually designed socket and corresponding liner to ensure optimal fit and transfer of force required for walking with the artificial leg. Apart from problems related to force transmission, socket-related problems such as skin irritations ranging up to ulcerations, excessive sweating, time-consuming donning, and doffing are commonly noted [for a review, see other studies (5–9)]. During the last two to three decades, transcutaneous bone-anchored osseointegrated prosthesis systems (TOPS) have become a reliable alternative for rehabilitation, especially for patients with recurrent problems associated with the socket (7–11). While the risk of recurrent infections due to the transcutaneous implant turned out to be much lower than expected, switching from socket prosthesis to a bone-anchored one is associated with numerous advantages ranging from improved rehabilitation and autonomous mobility up to the restoration of osseoperception (7, 8, 10, 11). Apart from differences regarding the geometry, the retention as well as the bone- and skin-implant interfaces, and the abutment for connection of the exoprosthesis, the principle of function is always the same (12).

### Case report

**Figure 1:**
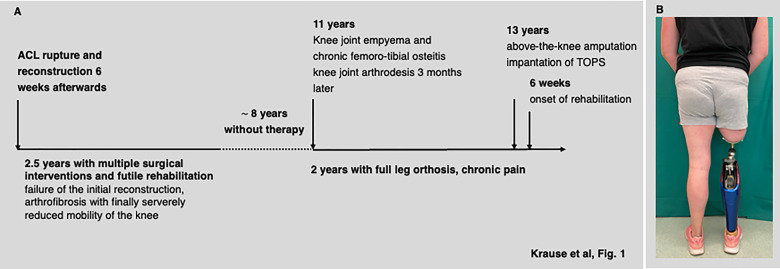
History of disease (**A**) and a photograph of the patient 18 months after switching to transcutaneous osseointegrated prosthesis system (TOPS) (**B**).

We report on a case of a 31-year-old woman who presented with a knee joint arthrodesis and an almost non-functioning leg, impeding her mobility and the care for her two children, ages 2 and 4 years, respectively. Clinical work-up revealed a 13-year history (Figure 1A) of recurrent failure following ACL reconstruction and numerous surgical interventions due to recalcitrant deep joint infection ending up with a knee joint arthrodesis *via* an Ilizarov circular frame (Figure 1A). Despite proven osseous fusion of the arthrodesis employing CT scanning, the patient suffered from immobilizing pain making a permanent medication with opioids necessary. In addition, signs and symptoms of early degenerative joint disease were already present in the contralateral hip. She was pretty well-informed on the rehabilitative power of a transfemoral amputation, especially when combined with a bone-anchored prosthesis. Given the clinical findings and the explicit wish of the patient, an elective transfemoral amputation with concomitant implantation of a bone-anchored prosthesis as well as a panel of follow-up examinations to evaluate rehabilitation and outcome even in the perception of the patient was consented (Table 1).

**Table 1:** Time schedule of the examinations.

	Prior to surgery	Postoperative		
		6 months	9 months	18 months
MRI of the thighs	✕		✕	
Clinical gait analysis	✕	✕	✕	✕
SIGAM and K-level		✕		
AMPPRO		✕	✕	✕
Fall Risk Index		✕	✕	✕
LCI-5		✕	✕	✕

### Surgical procedures and supportive treatment

The patient received preoperative routine antibiotic prophylaxis with a third-generation cephalosporin 30 min before induction of general anesthesia. The dysfunctional leg was dissected 25 cm below the greater trochanter, at a level determined by the length and diameter of the implant. During the preparation of the residual femur, multiple tissue samples were taken from the intramedullary canal for microbiological testing, and all of them scored negative. The femoral stem (Eska Orthopaedics, Lübeck, Germany) with a size of 15 mm × 180 mm (diameter × length) was driven into the medullary canal, and a tight press-fit was achieved (7). The muscles of the thigh were cut and shaped to avoid perspective interference with the prosthetic stem adaptor. The postoperative course was uneventful and, most interestingly, the patient was free of pain almost immediately after surgery in that opioids could be tapered to zero within less than 4 weeks. About 6 weeks after the first surgery the stoma of the TOPS was created using a specific circular punch. The skin-perforating bridging connector was attached to the femoral component essentially as described (7) and conventional means of postsurgical analgesics were employed. Starting the second postoperative day, the patient was instructed about daily self-care of the stoma, that is, to clean it twice daily with mild soap and water (7). Furthermore, she was equipped with a prosthesis, consisting of a microprocessor-controlled knee (Genium ×3, Otto Bock, Duderstadt, Germany) with a low-weight prosthetic foot (Taleo, Otto Bock, Duderstadt, Germany). She was encouraged to start walking with crutches to ensure partial weight-bearing of initially 5–10 kg. Full weight-bearing and walking without crutches were allowed about 6 weeks after the second surgery. Physiotherapy, gait, and balance training were initiated once she had received the prosthesis and was continued for about 4 months in order to restore her walking abilities (Figure 1B).

### Clinical gait analysis and questionnaires

For standardized clinical gait analysis, the GRAIL (Gait Real-time Analysis Interactive Lab; Motekforce Link, Amsterdam) was used essentially as described and the same immersive virtual reality was presented throughout the examinations (13). Per examination, the patient was allowed to familiarize herself by walking for at least 5 min at a self-selected comfortable speed. Subsequently, spatio-temporal parameters of gait together with kinematic and kinetic data were recorded during a 30-s interval, representing at least 52 steps. Mean and standard deviation were calculated and for spatio-temporal parameters repeated measures, ANOVA (SPSS statistical package 25, SPSS Inc. Chicago, Illinois, USA) was employed to assess longitudinal changes. Similarly, the mean of kinetic and kinematic data were plotted for longitudinal comparison.

Restoration of walking abilities and autonomous mobility was characterized by the K-level, by the SIGAM grade (14, 15), the Amputee Mobility Predictor with the use of a prosthesis (AMPPRO) test (16), scoring of the Fall Risk Index (17), and the patient-reported Locomotor Capabilities Index (LCI-5) (18). Magnetic resonance imaging (1.5T Magnetom Avanto Fit, Siemens Healthineers, Germany) 9 months after the index procedure served as a tool to assess the muscle volumes of both thighs relative to the findings prior to surgery. In particular, the length of the region investigated corresponds to the length of the stump with the bottom edge of the tuber ischiadicum as an upper border. Transversal T1-weighted fat/water-separated sequences were recorded and a 3D slicer was employed for quantification (19).

## Results

### Outcome

The surgical procedures and postoperative recovery were uneventful and already at the time of the index procedure opioids could be tapered to zero. The artificial leg was coupled to the bone-anchored prosthetic stem the second day after the creation of the stoma, and since then, the patient followed a supervised balance and gait training to restore walking abilities.

**Figure 2:**
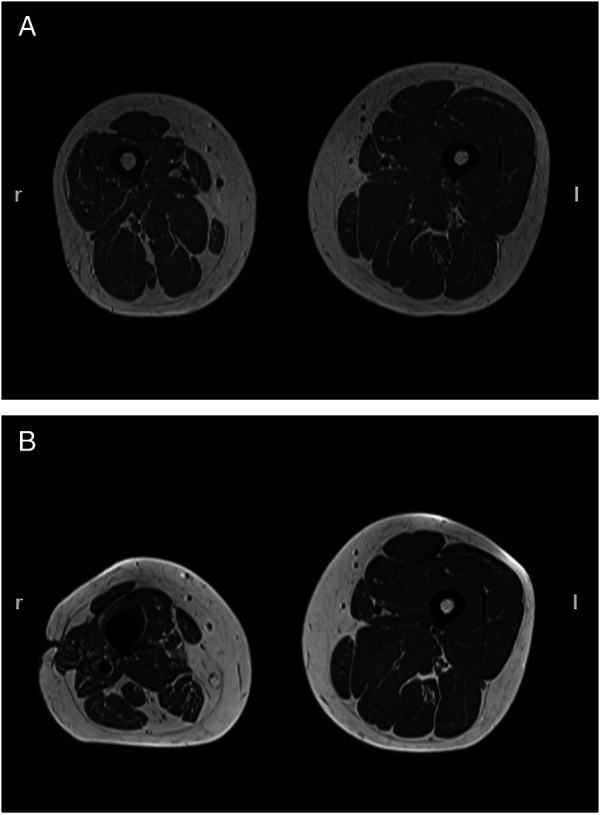
Transverse MRI of both thighs taken prior to transfemoral amputation (**A**) and 9 months after (**B**). Muscle volumes of the right (affected) thigh were 1,781 and 1,668 cm^3^, while those at the corresponding contralateral side were 2,750 and 3,378 cm^3^, respectively.

Magnetic resonance imaging confirmed severe atrophy of the thigh before the index procedure with muscle volumes of 1,781 cm^3^ on the affected side and 2,750 cm^3^ on the non-affected side in the corresponding thigh section. While 9 months after surgery the muscle volume of the affected thigh was almost the same as before, a 23% increase on the contralateral side was noted (Figure 2).

### Clinical gait analysis

**Figure 3:**
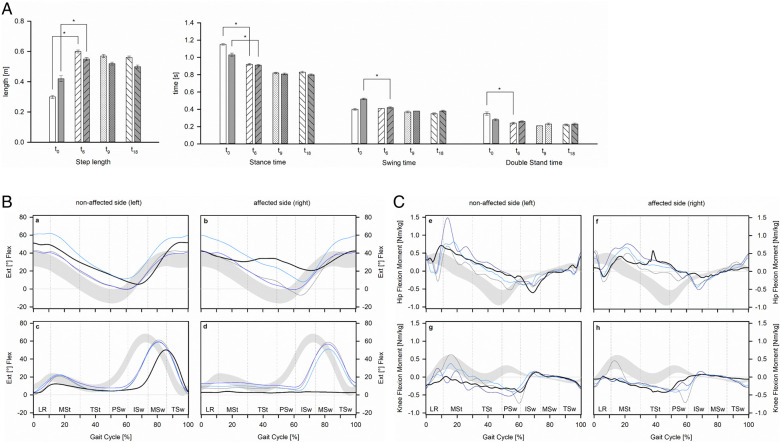
Spatio-temporal parameters of gait (**A**), kinematics (**B**), and kinetics (**C**) of hip and knee joints prior to transfemoral amputation and during the follow-up period. (**A**) Open and filled bars represent the non-affected and affected side prior to surgery (*t*_0_) as well as 6 (*t*_6_), 9 (*t*_9_), and 18 (*t*_18_) months after. (**B**) Kinematics of the hip (a,b) and knee joints (c,d) together with the normal range [gray area (24)] are presented prior to transfemoral amputation (black) as well as 6 (light blue), 9 (gray), and 18 (dark blue) months after switching to transcutaneous osseointegrated prosthesis system (TOPS). (**C**) Kinetics of the hip (e,f) and knee joints (g,h) together with the normal range [gray area (24)] are presented prior to transfemoral amputation (black) as well as 6 (light blue), 9 (gray), and 18 (dark blue) months after switching to TOPS. Vertical lines indicate the phases of the gait cycle, that is, LR, loading response; MSt, mid stance; TSt, terminal stance; PSw, pre-swing; ISw, initial swing; Msw, mid-swing; TSw, terminal swing (25).

Standardized clinical gait analysis confirmed severe limping and seriously impaired walking abilities at the time of the first presentation (Figure 3). Beyond a rather short stride length and an overall low walking speed kinetic and kinematic features deviated markedly from normal. This holds especially true for the non-affected side and might be taken as an indicator of the increased risk for degenerative joint disease and subsequent aggravation of here already poor mobility. Already 6 months after switching to TOPS, the spatio-temporal characteristics of gait were significantly improved and these changes persisted during the follow-up period. In particular, walking speed was almost doubled (0.50 ± 0.00 m/s at baseline vs. 0.96 ± 0.00 m/s at the time of the last follow-up examination; *p* < 0.001). The cadence (steps per minute) increased from 38.6 ± 0.37 at baseline up to 50.9 ± 0.48 (*p* < 0.001) and the stride time dropped (1.55 ± 0.01 s at baseline vs. 1.18 ± 0.01 s; *p* < 0.001), while the stride length increased from 0.77 ± 0.03 m to 1.13 ± 0.01 m (*p* < 0.001) and the stride width remained rather constant (data not shown). Contrasting to an almost immediate normalization of the spatio-temporal features of gait (Figure 3A) and the diminished differences between the affected and non-affected leg, the continuous adaptation of the kinetic and kinematic parameters to the new situation was seen (Figures 3B,C). In particular, kinematic and kinetic data from the non-affected hip and knee joints steadily reverted to the physiological pattern.

The patient achieved K-level type 3 and a SIGAM mobility grade F corresponding to a near normal gait ability within 18 months postoperatively (14, 15). Likewise, the patient achieved 41, 44, and 44 points out of 47 points with the AMPPRO, 26, 28, and 27 points out of 28 points with the Tinetti test, and 47, 54, and 54 points out of 56 points with the LCI-5 at the follow-up examinations 6, 9, and 18 months after the index procedure.

## Discussion

This case is two-fold unique in that we decided on a TOPS rather than the classical socket prosthesis and performed the first step of the TOPS procedure at the time of transfemoral amputation. Although there were serious concerns for infections due to the transcutaneous metal implant, these are mostly superficial and restricted to the stoma rather than ending with explantation of the prosthesis. Similarly, revision due to failure of the implant is rarely seen (7, 8, 10, 20–23).

At the time of her first presentation, she was seriously disabled with very limited autonomous mobility and chronic pain. Furthermore, she had been rather busy gaining knowledge on the pros and cons of the socket-mounted and bone-anchored prosthesis as measures to restore autonomous mobility after transfemoral amputation. Given the rather long history of the disease, she was not willing to try a socket prosthesis first, as she was seeking for a solution to her issues rather than for replacement of the existing problems with new ones. Thus, we followed her dedicated wish and paralleled the treatment and rehabilitation course with additional examinations. In particular, standardized clinical gait analysis prior to surgery confirmed severe limping with abnormal kinetics and kinematics for both legs. In other words, the risk for degenerative joint disease on the non-affected side and subsequent aggravation of here already poor mobility is foreseeable unless the dysfunctionality of the affected leg is solved. Already 6 months after switching to TOPS, a steep improvement in her walking abilities with diminished differences between the spatio-temporal features related to the affected and non-affected leg was noted, and these changes persisted during the follow-up period. The continuous normalization of kinematic and kinetic data especially on the non-affected side has to be considered as an important prerequisite to prevent the progression of degenerative joint disease. The restoration of bilateral symmetry by mutual approximation to the standard gait pattern during the 18 months of follow-up period mirrors her constant efforts to train and improve her walking abilities.

## Conclusion

Although the decision for unilateral transfemoral amputation is hard to achieve and requires careful counseling, there is no doubt that this procedure has the power to improve quality of life and autonomous mobility rather than worsening the situation. The outcome of our patient is well in line with this notion. Furthermore, a reasonable number of patients claim recurrent problems at the interface between stump and standard socket prosthesis, ranging from intradaily fluctuations of the stump volume to ulcera caused by relative movements of the socket (or liner) and the stump. Such issues may lead to fitting and re-fitting of the socket over and over and this we probably spared our patient. Nevertheless, long-term follow-up is necessary to detect future complications of TOPS.

## Data Availability

The original contributions presented in the study are included in the article/Supplementary Material, further inquiries can be directed to the corresponding author/s.
